# Surveillance strategies using routine microbiology for antimicrobial resistance in low- and middle-income countries

**DOI:** 10.1016/j.cmi.2021.05.037

**Published:** 2021-06-07

**Authors:** Cherry Lim, Elizabeth A. Ashley, Raph L. Hamers, Paul Turner, Thomas Kesteman, Samuel Akech, Alejandra Corso, Mayfong Mayxay, Iruka N. Okeke, Direk Limmathurotsakul, H. Rogier van Doorn

**Affiliations:** 1 Mahidol Oxford Tropical Medicine Research Unit, Faculty of Tropical Medicine, Mahidol University, Bangkok, Thailand; 2 Centre for Tropical Medicine and Global Health, Nuffield Department of Medicine, University of Oxford, Oxford, UK; 3 Lao-Oxford-Mahosot Hospital Wellcome Trust Research Unit, Vientiane, Laos; 4 Eijkman-Oxford Clinical Research Unit, Jakarta, Indonesia; 5 Cambodia Oxford Medical Research Unit, Angkor Hospital for Children, Siem Reap, Cambodia; 6 Oxford University Clinical Research Unit, National Hospital for Tropical Diseases, Hanoi, Viet Nam; 7 KEMRI-Wellcome Trust Research Programme, Nairobi, Kenya; 8 National/Regional Reference Laboratory for Antimicrobial Resistance (NRL), Servicio Antimicrobianos, Instituto Nacional de Enfermedades Infecciosas ANLIS Dr. Carlos G. Malbrán, Buenos Aires, Argentina; 9 Institute of Research and Education Development (IRED), University of Health Sciences, Vientiane, Laos; 10 Department of Pharmaceutical Microbiology, Faculty of Pharmacy, University of Ibadan, Ibadan, Nigeria

**Keywords:** Antimicrobial resistance, Drug-resistant infections, Low- and middle-income countries, Routine microbiology, Surveillance

## Abstract

**Background:**

Routine microbiology results are a valuable source of antimicrobial resistance (AMR) surveillance data in low- and middle-income countries (LMICs) as well as in high-income countries. Different approaches and strategies are used to generate AMR surveillance data.

**Objectives:**

We aimed to review strategies for AMR surveillance using routine microbiology results in LMICs and to highlight areas that need support to generate high-quality AMR data.

**Sources:**

We searched PubMed for papers that used routine microbiology to describe the epidemiology of AMR and drug-resistant infections in LMICs. We also included papers that, from our perspective, were critical in highlighting the biases and challenges or employed specific strategies to overcome these in reporting AMR surveillance in LMICs.

**Content:**

Topics covered included strategies of identifying AMR cases (including case-finding based on isolates from routine diagnostic specimens and case-based surveillance of clinical syndromes), of collecting data (including cohort, point-prevalence survey, and case—control), of sampling AMR cases (including lot quality assurance surveys), and of processing and analysing data for AMR surveillance in LMICs.

**Implications:**

The various AMR surveillance strategies warrant a thorough understanding of their limitations and potential biases to ensure maximum utilization and interpretation of local routine microbiology data across time and space. For instance, surveillance using case-finding based on results from clinical diagnostic specimens is relatively easy to implement and sustain in LMIC settings, but the estimates of incidence and proportion of AMR is at risk of biases due to underuse of microbiology. Casebased surveillance of clinical syndromes generates informative statistics that can be translated to clinical practices but needs financial and technical support as well as locally tailored trainings to sustain. Innovative AMR surveillance strategies that can easily be implemented and sustained with minimal costs will be useful for improving AMR data availability and quality in LMICs.

## Introduction

Surveillance of antimicrobial resistance (AMR) is a fundamental action to inform efforts to control the spread of AMR through generation of evidence for local, national and global guidance and action plans [1]. The main objectives of AMR surveillance include (a) providing local evidence for empirical treatment guidelines and clinical decision-making, (b) benchmarking to assess the effect of stewardship interventions, (c) estimating burden for epidemiological purposes, and (d) tracing differences and changes in space and time including outbreak detection [1,2].

Various AMR surveillance modalities have been described by the World Health Organization (WHO) [3]. Surveillance can be comprehensive (the entire population at risk) or sentinel (limited catchment area); continuous, episodic or periodic; passive (using routinely collected data) or active (planned collection of data otherwise unavailable); routine (systematic collection of data on regular basis); or enhanced (collection of additional data) [3—6]. These distinctions are important to standardize data flow of surveillance across time and space, and more importantly to guide collection of quality data [2,3]. In both low- and middle-income countries (LMICs) and in high-income settings, where more laboratories are available and microbiology testing is more often performed as part of the standard of care, passive AMR surveillance using routine microbiology data of clinical isolates from sentinel sites is the most commonly used approach [7].

At sentinel sites, AMR surveillance involves collection, validation, interpretation and reporting of susceptibility data. Microbiological testing is a crucial tool for diagnosis of bacterial infections and a source of AMR data in LMICs. Optimizing existing tools for diagnosing bacterial infections and generating bacterial identification and antimicrobial susceptibility data on site is the foundation of an AMR surveillance system [8,9]. Potential approaches for strengthening laboratory systems and services in LMIC settings have previously been extensively discussed [8—12]. Here, we focus on different strategies for AMR surveillance in humans, using data from microbiology laboratories generated as part of standard of care. We will highlight potential strengths, weaknesses, systematic errors and biases that may arise at each stage of data acquisition, and identify areas where systematic support is needed to improve and maintain the quality of AMR data from LMICs.

## Methods

PubMed was searched for literature published before 1st November 2020, with the main theme of AMR surveillance using routine microbiology data in LMICs as search terms. Titles, abstracts, and full texts of search results were reviewed (see Supplementary Material). Moreover, we reviewed the relevant citations and AMR reports that were mentioned in the key papers and the strategies used for identifying AMR. Identified strategies are summarized in Table 1.

### Strategies for identifying AMR/drug-resistant infections (DRIs)

There is an extensive body of literature on different strategies for identifying AMR/DRIs in a prespecified population using routine microbiology data [8,13]. In general, there are two main approaches to identifying cases of DRI: case-finding based on routinely collected clinical diagnostic specimens, and case-based surveillance of clinical syndromes (Table 1) [14].

#### Case-finding based on routine diagnostic specimens

This approach was described in the WHO Global AMR Surveillance System (GLASS): cases are identified through microbiology results from routinely collected clinical diagnostic specimens [14]. This method is easy to implement when sentinel sites have a microbiology laboratory, microbiologists and technicians, and the data are available. Often, targeted pathogens from priority specimens are surveyed, and sterile compartments such as blood and cerebrospinal fluid are the primary choice of sample owing to their higher specificity in representing true infections [2,15,16]. Combined with epidemiological and clinical data (i.e. origin of infection, age, and underlying illness), this approach can provide informative data. For instance, GLASS aims to report the frequency of infections caused by pathogens non-susceptible to individual defined antibiotics under surveillance per infection origin (community or hospital) and specimen (blood and urine) using routine microbiology data and epidemiology data collected from sentinel sites, although not all enrolled countries provide sufficient data [17]. Surveillance of stool samples collected for routine diagnostic purposes from patients with diarrhoea has been performed to monitor resistance among pathogens such as *Salmonella* spp [18]. Alternatively, a less preferable approach is the utilization of microbiology data alone (laboratory-based surveillance) without clinical and epidemiological data. Most national and supranational AMR networks rely solely on laboratory data to trace changes of antimicrobial susceptibility in space and time [7,19—22]. A recent review on supranational networks performing AMR surveillance in LMICs showed the heterogeneity of data due to lack of standards on the composition and activities of surveillance networks [7], and the majority (80%, 39/49) describe AMR based on samples sent to laboratory for clinical purposes (Table 2). Moreover, several of these supranational networks are led by pharmaceutical companies and involve collection of isolates with specific objectives (e.g. evaluating susceptibility to new antibiotics or antibiotics under development) [7]; thus use of those data for surveillance needs to be done with caution. Overall, the strategy of case-finding based on routinely collected clinical diagnostic specimens has been the most commonly used approach and has been shown useful to estimate the burden of AMR and the resistant proportions of important pathogens in studies in LMICs [23,24].

A potential concern of AMR surveillance based on routine microbiology data alone is the representativeness of data for the target population, especially when microbiological diagnosis is not performed systematically and important parameters are not captured. First, diagnostic microbiology testing may be systematically underused (i.e. patients with infectious diseases are treated with empiric antibiotics without blood culture testing) and selectively delayed (i.e. patients who have failed empiric treatments are more likely to be sampled for microbiological testing) in LMICs [25,26]. Consequently, the incidence of DRIs is underestimated and the proportion of AMR overestimated [25,27—29]. Second, in cases when the decision to collect specimens for microbiological testing is based on physicians’ subjective judgement and practices vary by physician and hospital, or are impacted by patients’ ability to afford costs for testing, difficulties in data interpretation and comparability across time and space are expected. There are several reasons for underuse of microbiology in LMICs: the costs of prescribing antibiotics may be lower than performing microbiology testing; results take too long while antibiotics may have to be given immediately [31]; and the microbiology laboratory often returns negative culture results and this is viewed as a waste of resources with minimal returns to benefit patients. There can be an absence of financial support systems or insurance to cover the costs of diagnosis. The laboratory infrastructure may be poor or outdated [32], weak supply chains may lead to stockouts in systems and affect consistency and availability; there can be a lack of trust in the results often due to a lack of qualified microbiologists and laboratory technicians, and issues with the quality and communication of results [27,30]; lastly, in many LMICs, the costs of reagents and consumables, equipment and maintenance for the microbiology laboratory can be double that of high-income settings. Absence of local production of essential supplies imposes additional costs related to customs services and annual inflation.

#### Case-based surveillance of clinical syndromes

This approach is based on patients who present for medical care with signs and symptoms of infection. Compared to the laboratory-based approach, the case-based approach is more robust to variations in the use of microbiology and selection bias, as case identification and specimen collection time is based on more systematic and objective data collection [13,14]. Moreover, supported by clinical data, microbiology results of samples other than blood and cerebrospinal fluid can be used more effectively. The majority of supranational AMR surveillance networks that are based on case-based surveillance are currently targeted at sepsis, meningitis, and pneumonia (Table 2).

A key advantage of case-based surveillance is that the data can be used to support clinical decision-making and choice of empiric antibiotics [13,33,34]. The denominator of the data is the total number of patients with a specific type of infection or condition (i.e. patients with central lines, with community- or hospital-acquired infections) [13,14]. An example of the use of such data for empirical treatment is the weighted-incidence syndromic combination antibiogram (WISCA) that reports antimicrobial sensitivity testing (AST) data by suspected infections [33]. The numerator is the number of patients by infection site (e.g. urinary-tract infection) and by each pathogen that is susceptible to at least one of the agents in a given regimen (e.g. meropenem combined with vancomycin), and the denominator is the total number of patients by infection site with available antibiogram data for the full antibiotic regimen [33].

Increasingly, case-based surveillance has been discussed [2,8,13] and adopted in LIMCs [34—38]. An example is ACORN (A Clinically Oriented antimicrobial Resistance surveillance Network), which actively identifies cases using clinical diagnosis/suspicion and actively collects clinical metadata alongside microbiology results [29].

There are challenges in implementing case-based surveillance in LMICs. First, comprehensive clinical data are not available in electronic format in most LMIC settings. Clinical data are important for defining infections, but routine documentation of such data and the use of accepted case definitions can be challenging in LMICs. The challenges include a low ratio of healthcare-workers to patients, lack of supporting tests (e.g. radiology), and lack of diagnostic guidelines to rapidly differentiate bacterial from other infections. Second, there is a lack of trained personnel and guidelines in local languages to support case-based surveillance. Third, there is a lack of open-access, easy-to-use information technology to support clinical data capturing and storage and often limited exchange of clinical information between clinicians and the laboratory which may impact the quality/relevance of laboratory results [34]. Fourth, similar to laboratory-based surveillance, incidence of DRIs (i.e. the number of infections caused by resistant bacteria per 100 000 population) can be underestimated when there is underuse of routine microbiology testing.

### Survey designs

Multiple survey designs have been used to collect AMR data in hospitals in LMICs. These include cross-sectional point-prevalence surveys (PPSs), observational cohorts, and case—control designs. PPSs have been used to measure the prevalence of healthcare-associated infections and antimicrobial use and resistance in healthcare settings in LMICs [37,39,40]. In PPS studies, patients are actively screened and identified using a case-based surveillance approach, hence the design often generates representative data that allow high comparability in space and time. The challenge of PPSs is that they require teams of well-trained staff to screen cases. Observational cohort studies using case-finding based on specimens routinely sent to laboratories for clinical purposes have been done in LMICs and can easily be implemented using existing microbiology data [16,24,25,41]. Observational cohort studies using a case-based surveillance approach to prospectively enroll cases have been done in LMICs [36,38,42—44], but are challenging for a resource-limited setting, and are often performed as parts of research projects. The implementation of cohort studies in general is particularly difficult for healthcare-associated infections because of the large variations in the time to infection among the infected patients, and because a large time investment is often needed to identify a case. Nonetheless, when performed appropriately the data generated often have large potential value in supporting empiric treatment guidelines and answering epidemiological research questions. In Asia, case-based surveillance targeting high-risk populations, such as patients admitted to intensive care units or patients with central lines, have been conducted prospectively to provide baseline evidence for prevention and control of DRIs [38,45,46]. From the literature review, we have identified some case—control studies, and most of these studies had the objective of estimating the attributable mortality of AMR infections and risk factors for acquiring AMR infections [47—49].

### Sampling strategies

When population-based surveillance cannot be performed for practical reasons, various sampling approaches can be considered. Randomly sampled study cohorts and/or surveillance sites from a large target population is a common approach. Studies using lot quality assurance sampling (LQAS) have been shown to be useful in informing whether or not resistance of a pathogen to specific antibiotics is high, which is essential to inform the appropriateness of using those antibiotics for empiric treatment [50,51]. LQAS requires fewer resources and a lower budget compared to population-based surveillance, and has been shown to be feasible in LMICs [50].

The considerations for when sampling needs to be done are the sample size, sampling approach, and sampling frequency [3]. The key is to ensure the sample represents the target population as closely as possible. In LMICs, central and referral laboratories are overrepresented, as peripheral healthcare centres often lack the infrastructure to perform routine microbiology testing, thus limiting the availability of AMR data to support local clinical decisions and to contribute to national and global estimates of AMR burden.

When attempting to estimate the health impact due to AMR infections, various sampling strategies for comparator cohorts have been suggested. WHO GLASS recently published a guideline for estimating attributable mortality of AMR bloodstream infections (BSIs) [52], in which the recommendation is to collect data from cohorts of patients without an infection and with infections caused by drug-susceptible isolates to use as comparator groups [52]. A recommended strategy is to use the exposure density sampling with matching based on patient characteristics [52,53]. This approach ensures that the time-dependent exposure (i.e. occurrence of BSIs) can be appropriately adjusted for and can produce unbiased estimates with a more realistic interpretation than random selection methods [53,54]. Alternatively, a commonly used strategy to sample patients without an infection is by randomly selecting eligible patients (sometimes matching key confounders such as length of hospitalization prior to sample collection). However, the caveat of random selection is that the interpretation is often conditioned on a future event (i.e. under the assumption that comparator groups remain within the group throughout the entire period of hospitalization) [53,54].

### Strategies for processing and analysing data

At the global level, GLASS published protocols to standardize AMR surveillance data processing. In general, it is recommended that repeated isolates from individual patients are to be removed before estimating prevalence and incidence of AMR infections (deduplication), and data should be stratified by infection origin. Increasingly, open-access applications such as WHONET [55] and AMASS [56] are available to support local sentinel sites, where there is a lack of time and expertise, to process AMR surveillance data and to generate reports. It is acknowledged that there will be challenges in integrating these applications into routine practice of microbiology data collection and into the existing LIS at a local sentinel site. Innovative methods of processing and reporting AST profiles, including WISCA, have been shown to be useful but are so far not commonly applied in LMICs. Frameworks such as Microbiology Investigation Criteria for Reporting Objectively (MICRO) are useful to enhance reporting of microbiology data and comparability of AMR surveillance data, as well as providing some quality assurance [29].

At the national and global level, population catchment and pattern of healthcare utilization in the population are ideally to be included in AMR surveillance reports to interpret AMR surveillance data. However, such statistics are not commonly available in LMICs. The use of data from existing Health and Demographic Surveillance Sites (HDSS) to combine community- and hospital-based clinical and microbiology data has been recommended [8,57] but has not been sufficiently used in LMICs. An example of an HDSS network is the INDEPTH network, from which data have been utilized to estimate mortalities from malaria [58] and HIV/AIDS [59] in Africa and Asia, as well as for comparing antibiotic access and use across different regions [60]; however, so far this approach has not been used for DRIs [61].

### Support to increase use of microbiology testing

A paradigm shift in the value of microbiological testing and prioritization of the detection of drug-resistant infections is needed in many LMICs [9]. Innovative methods to improve quality and reduce the costs of microbiological testing are needed. Incentives—such as patient health insurance coverage and hospital reimbursement coverage to support the costs of microbiology testing—may support this shift and motivate the use of microbiology testing in LMIC settings. A healthy and dedicated liaison between the qualified microbiology laboratory and clinicians is also crucial to increase mutual trust and to optimize the use of existing diagnostic tools [62—64]. Moreover, a clear potential advancement in career pathways for the qualified microbiologist and laboratory technicians could further enhance trust and uptake of microbiology results.

While the use of microbiology in many LMICs is gradually increasing, essential parameters such as blood culture use rate and rates of AMR stratified by antibiotic usage should also be used to standardize the measures of prevalence and incidence, and increase the comparability of AMR burden across space and time [25].

Social and behavioural changes on the attitudes and practices of antibiotic usage and microbiological testing are needed and may be accelerated with diagnostic and antibiotic stewardship programmes. Systematic trainings to increase awareness on the significance of microbiology testing among all stakeholders, from students to senior specialists, may motivate the use of microbiology.

Investments in improving microbiology quality and use through expensive automated equipment do not always deliver, as operating costs and costs of maintenance and consumables have not been taken into account. Maintaining power supply and cold chain often remains challenging. Innovative tools to simplify microbiological diagnosis [65] and to conserve diagnostic reagents will be useful to fill the AMR data gap from LMIC settings.

### Support to establish and maintain case-based surveillance

The incremental value of the case-based approach can be significant if implementation challenges can be overcome [13]. Trainings on diagnostic stewardship and pragmatic definitions of suspected bacterial infections would be important in LMIC settings. Devices, software and technical support to capture and store clinical data are needed. Most importantly, financial support is needed to build the local capacity, including establishing surveillance teams and increasing availability of diagnostic tools, in order to ensure a sustainable case-based surveillance system. Increasingly, different international organizations have devoted efforts to strengthening AMR surveillance in LMICs. An example is the Fleming Fund (UK government), which supports 24 LMICs in Africa and Asia [66].

### Support to strengthen laboratory quality and microbiology data quality

Quality assurance and control (QA/QC) are essential to ensure accurate identification of pathogens and AST profiles, and need to be improved in sentinel surveillance sites in LMICs. Commercial QA/QC schemes and accreditation programmes are available, but often unaffordable for laboratories in LMICs. Training and support to ensure quality performance and methods of bacterial identification and AST are needed for laboratories in LMICs [67]. Web-based tools such as the laboratory quality stepwise implementation tool and other quality management-strengthening programmes, are useful to guide laboratories, especially those in LMIC settings, towards implementing quality management systems [68—70]. In addition, prioritizing the microbiology testing in the local setting, increasing the number of microbiology testing facilities, and strengthening the local capacity of the existing laboratory system are needed.

Verifying AST results of bacterial isolates is an important component of surveillance to ensure quality data are generated. However, data verification and highlighting isolates with unusual AST results can be a complicated and time-consuming process to perform manually. Commercial laboratory information management systems with functions to support microbiology data verification are unaffordable for many hospitals in LMICs. The openaccess WHONET programme has functions to support data quality checks [55]. Open-access, offline, and user-friendly tools that can automatically process and analyse microbiology data can be useful in resource-limited settings in generating AMR surveillance reports for local use and sharing in a timely fashion.

Finally, there is a lack of evaluation frameworks for continuous AMR surveillance systems in LMICs. Regular evaluations on the performance of AMR surveillance systems in LMICs would be useful to identify limitations and areas for improvement as well as to quantify and qualify progress when it occurs. Examples of evaluation tools for AMR surveillance network are the AMR Progressive Management Pathway tool developed by the Food and Agriculture Organization of the United Nations, NEOH and SURV-TOOLS [71]. The implementation of such tools in LMIC settings is uncommon.

## Conclusion

Several strategies for conducting AMR surveillance have been used in LMICs, with the majority adopting the isolate- or samplebased approach using microbiology data from routine clinical diagnostics. Attempts to transition to a case-based approach are receiving increased attention, but significant challenges remain in settings where resources, expertise and experience are limited. Guidelines, trainings, and local capacity building are useful to support such transitions. Moreover, tertiary hospitals, research institutions, and pharmaceutical companies with microbiology testing and involved in AMR data collection could play a role in leading and supporting the transition to case-based AMR surveillance in surrounding facilities within their reach. In parallel, it is important to strengthen diagnostic capacity, reduce costs and otherwise incentivize routine microbiology testing, especially when most surveillance still relies on a laboratory-based approach in central reference hospitals. Strategies to maximize usage of locally existing resources and data could narrow the data gap. Improvements in AMR data generated from LMICs also depends on innovative and collaborative clinical research on strategies that can require minimal financial and human resource requirements while data quality is maintained. For instance, the LQAS has shown applicability in LMICs to inform empirical antibiotic treatment guidelines in a local setting, and WISCA could be an informative strategy for presenting AMR patterns to guide treatment decisions. Experience from ACORN implementation and further roll-out will inform what is needed for this transition in LMICs. Finally, innovative AMR surveillance tools and strategies that can be easily implemented and maintained in resource-limited settings will be useful for improving AMR data availability and quality to support local, national and global action plans in controlling AMR.

## Supplementary Material

Supplementary data to this article can be found online at https://doi.org/10.1016/j.cmi.2021.05.037.

Supplementary data

## Figures and Tables

**Table 1:** Summary on strength and weakness of various strategies for antimicrobial resistance (AMR) surveillance

	Strength	Weakness
**Strategies for identifying AMR infections:**		
Case-finding based on specimens sent routinely to laboratories for clinical purposes	- Relatively easy to implement and sustain in LMICs - Generate basic data when only laboratory data is available - Generate informative data (e.g. origin of infection) when epidemiological and clinical data (e.g. hospital admission date) are also available - Generate useful statistics including proportion of samples with growth of non-susceptible bacteria of the species and antibiotic under surveillance per specimen type; proportion of sampled patients with positive culture of any pathogenic bacteria per specimen type (in the cases when data on negative growth is available); and frequency of patients with growth of non-susceptible bacteria per specimen type, species and antibiotic [2] - Capable of generating data for outbreak detection, but potential influences on the data due to the use of microbiology testing and empirical antibiotic prescription behaviour should considered carefully	- Estimates on incidence of infection and proportion of AMR can be influenced by utilization of microbiology testing and empiric antibiotic prescription behaviour - Comparability across space and time is often limited in LMIC settings - Capability of providing local evidence for empiric treatment guidelines and clinical decision-making is limited, especially in cases when there is a lack of clinical data
Case-based surveillance of clinical syndromes	- Relatively robust to variations in use of microbiology testing as case definitions allow more systematic and objective data collection - Informative data can be generated to inform clinical decisions - Capable of addressing different objectives of AMR surveillance including (a) providing local evidence for empiric treatment guidelines; (b) benchmarking to assess the effect of stewardship interventions; (c) estimating health impact of AMR infection; and (d) tracing differences and changes in space and time	- Can be labour-intensive and costly to implement and sustain in LMICs - Needs investments on training, guidelines, and diagnostic capacity in LMIC settings
**Sampling strategies:**		
DRI: include consecutive samples	- Easy to perform	- At risk of bias due to clinical sampling behaviour
DRI: lot quality assurance sampling (LQAS)	- Requires small sample size for informative estimates to inform empiric treatment policy	- Definition of thresholds defining the ‘low’ or ‘high’ prevalence of resistance could be challenging to determine
Comparator cohort: exposure density sampling	- Ensures a more accurate estimation for health burdens due to DRI	- Would need training and detailed protocol for LMIC settings
**Strategies for reporting AST data:**		
Report susceptibility to individual antibiotic	- Easy to generate the statistics	- Limited capability in translating to clinical practice
Weighted-incidence syndromic combination antibiogram (WISCA)	- Statistics generated can be translated to clinical practice	- May be difficult to generate in LMICs where there is a lack of expert and open-access applications to process data

AST, antibiotic susceptibility testing; DRI, drug-resistant infection; LMIC, low- and middle-income countries.

**Table 2:** Strategies for identifying antimicrobial-resistant infections used by antimicrobial resistance (AMR) surveillance networks (a list adapted from Ashley et al., 2018 [7], stratified by strategy used and arranged in alphabetical order) in low- and middle-income countries (LMICs)

Name	Year	Target infections/organisms	Strategy used to identify AMR cases
A Clinically Oriented Antimicrobial Resistance Surveillance Network (ACORN)	2019 ongoing	Sepsis; meningitis; pneumonia (both community-acquired and hospital-acquired)	Case-based surveillance of clinical syndrome
Antimicrobial Resistance Epidemiological Survey on Cystitis (ARESC), European Society for Infection in Urology	2003—2006	Uncomplicated cystitis	Case-based surveillance of clinical syndrome
Bacterial Infections and Antibiotic-Resistant Diseases Among Young Children in Low-Income Countries (BIRDY), Institut Pasteur International Network	2012 ongoing	Sepsis; meningitis; pneumonia	Case-based surveillance of clinical syndrome
Burden of Antibiotic Resistance in Neonates from Developing Societies	2015—2018	Neonatal sepsis	Case-based surveillance of clinical syndrome
Clinical Information Network—Antimicrobial Resistance (CINAMR) project	2021—2023^a^	A project that may feed data into other initiative such as ACORN network and WHO GLASS	Case-based surveillance of clinical syndrome
Diseases of the Most Impoverished Typhoid Study Group and Multicentre Shigellosis Surveillance Study (DOMI), International Vaccine Institute, Republic of Korea	2001 —2004	Typhoid fever	Case-based surveillance of clinical syndrome
Global Point Prevalence Survey of Antimicrobial Consumption and Resistance (Global-PPS), University of Antwerp	2015 ongoing	Hospital-acquired infections	Case-based surveillance of clinical syndrome
Hib Impact Project (Pediatric Bacterial Meningitis Surveillance Network)	2006—2008	Meningitis	Case-based surveillance of clinical syndrome
International Nosocomial Infection Control Consortium (INICC)	2002 ongoing	Clinically defined pneumonia; laboratory-confirmed bloodstream infection; clinical sepsis; symptomatic urinary tract infection	Case-based surveillance of clinical syndrome
Proof-of-Principle routine diagnostics project for antimicrobial resistance surveillance (PoP project), CAESAR	2018 ongoing	Suspected bloodstream infections	Case-based surveillance of clinical syndrome
South Asian Pneumococcal Alliance (SAPNA), GAVI Alliance	2004—2009	Sepsis; meningitis; pneumonia (children 2—5 years old)	Case-based surveillance of clinical syndrome
Surgical Unit-based Safety Programme (SUSP)	2013—2015	Surgical site infection	Case-based surveillance of clinical syndrome
The Alexander Project, GlaxoSmithKline	1992—2002	Bacteria	Case-finding based on specimens sent to laboratory for clinical purposes
Asian Network for Surveillance of Resistant Pathogens (ANSORP), Sungkyunkwan University, Korea	1996 ongoing	Bacteria	Case-finding based on specimens sent to laboratory for clinical purposes
Antibiotic Resistance in the Mediterranean Region (ARMed), Infection Control Unit, Mater Dei Hospital, Msida, Malta	2003—2007	Bacteria	Case-finding based on specimens sent to laboratory for clinical purposes
ARTEMIS Global Antifungal Surveillance Programme (ARTEMIS)	1997—2005	Fungi	Case-finding based on specimens sent to laboratory for clinical purposes
Assessing Worldwide Antimicrobial Resistance and Evaluation Programme (AWARE), International Health Management Associates, Inc. (IHMA)	2012 ongoing	Bacteria	Case-finding based on specimens sent to laboratory for clinical purposes
Central Asian and Eastern European Surveillance of Antimicrobial Resistance (CAESAR)	2013 ongoing	Bacteria	Case-finding based on specimens sent to laboratory for clinical purposes
Caribbean Public Health Agency (CARPHA)	2013 ongoing	Bacteria	Case-finding based on specimens sent to laboratory for clinical purposes
Community-Acquired Respiratory Tract Infection Pathogen Surveillance (CARTIPS)	2009—2010	Bacteria	Case-finding based on specimens sent to laboratory for clinical purposes
Centre for Disease Dynamics, Economics and Policy (CDDEP)/ResistanceMap	1999 ongoing	Bacteria	Case-finding based on specimens sent to laboratory for clinical purposes
Community-Based Surveillance of Antimicrobial Use and Resistance in Resource-Constrained Settings, WHO	2002—2005	Bacteria	Case-finding based on specimens sent to laboratory for clinical purposes
Comparative Activity of Carbapenem Testing (COMPACT and COMPACT II), Janssen Asia-Pacific	2008—2010	Bacteria	Case-finding based on specimens sent to laboratory for clinical purposes
International Daptomycin Surveillance Programmes, JMI Laboratories	2011 ongoing	Bacteria	Case-finding based on specimens sent to laboratory for clinical purposes
European Antimicrobial Resistance Surveillance Network (EARS-Net), ECDC	1999 ongoing	Bacteria	Case-finding based on specimens sent to laboratory for clinical purposes
Enter-Net International Surveillance Network, Health Protection Agency, UK	1993—2007	Bacteria	Case-finding based on specimens sent to laboratory for clinical purposes
Food- and Waterborne Diseases and Zoonoses Network (FWD-Net), ECDC	2007 ongoing	Bacteria	Case-finding based on specimens sent to laboratory for clinical purposes
Gonococcal Antimicrobial Surveillance Programme (GASP), WHO	1992 ongoing	Bacteria	Case-finding based on specimens sent to laboratory for clinical purposes
International Network For Optimal Resistance Monitoring (INFORM), IHMA	2012—2014	Bacteria	Case-finding based on specimens sent to laboratory for clinical purposes
International Network for the Study and Prevention of Emerging Antimicrobial Resistance (INSPEAR), US CDC	1998—2010	Bacteria	Case-finding based on specimens sent to laboratory for clinical purposes
In Vitro Activity of Oral Antimicrobial Agents Against Pathogens Associated With Community-Acquired Upper Respiratory Tract and Urinary Tract Infections: A Five Country Surveillance Study, IHMA	2012—2013	Bacteria	Case-finding based on specimens sent to laboratory for clinical purposes
Multiyear, Multinational Survey of the Incidence and Global Distribution of MBL-Producing Enterobacteriaceae and Pseudomonas aeruginosa, IHMA	2012—2014	Bacteria	Case-finding based on specimens sent to laboratory for clinical purposes
Minocycline activity tested against *Acinetobacter baumannii* complex, *Stenotrophomonas maltophilia* and *Burkholderia cepacia* species complex isolates from a global surveillance programme (2013), JMI Laboratories	2013	Bacteria	Case-finding based on specimens sent to laboratory for clinical purposes
Meropenem Yearly Susceptibility Test Information Collection (MYSTIC), AstraZeneca	1997—2008	Bacteria	Case-finding based on specimens sent to laboratory for clinical purposes
Mortality from Bacterial Infections Resistant to Antibiotics (MBIRA)	2020 ongoing	Gram-negative enteric bacteria	Case-finding based on specimens sent to laboratory for clinical purposes
NosoMed Pilot Survey in the Eastern Mediterranean Area, Universite Claude Bernard Lyon I	2003—2004	Bacteria	Case-finding based on specimens sent to laboratory for clinical purposes
Programme to Assess Ceftolozane/Tazobactam Susceptibility (PACTS), Cubist Pharmaceuticals	2012 ongoing	Bacteria	Case-finding based on specimens sent to laboratory for clinical purposes
Pan-European Antimicrobial Resistance Using Local Surveillance (PEARLS), Wyeth Pharmaceuticals	2001—2002	Bacteria	Case-finding based on specimens sent to laboratory for clinical purposes
Prospective Resistant Organism Tracking and Epidemiology for the Ketolide Telithromycin (PROTEKT), Sanofi-Aventis	1999—2004	Bacteria	Case-finding based on specimens sent to laboratory for clinical purposes
Red Latinoamericana de Vigilancia de la Resistencia a los Antimicrobianos (ReLAVRA), PAHO	1996 ongoing	Bacteria	Case-finding based on specimens sent to laboratory for clinical purposes
Study on Antimicrobial Resistance in *Staphylococcus aureus*(SARISA), LEO Pharma (Copenhagen)	1996 ongoing	Bacteria	Case-finding based on specimens sent to laboratory for clinical purposes
SENTRY Antimicrobial Surveillance Programme, JMI laboratories	1997 ongoing	Bacteria, fungi	Case-finding based on specimens sent to laboratory for clinical purposes
Sistema de Redes de Vigilancia de los Agentes Responsables de Neumonias y Meningitis Bacterianas (SIREVA and SIREVA II), PAHO	1993—onging	Bacteria	Case-finding based on specimens sent to laboratory for clinical purposes
Study for Monitoring Antimicrobial Resistance Trends (SMART), Merck & Co. Inc.	2002—2011	Bacteria	Case-finding based on specimens sent to laboratory for clinical purposes
Survey of Antibiotic Resistance (SOAR), GlaxoSmithKline	2002 ongoing	Bacteria	Case-finding based on specimens sent to laboratory for clinical purposes
International Solithromycin Surveillance Programmes, JMI Laboratories, USA	2011 ongoing	Bacteria	Case-finding based on specimens sent to laboratory for clinical purposes
TARGETed Surveillance Study, GR Micro Ltd, UK	2003—2007	Bacteria	Case-finding based on specimens sent to laboratory for clinical purposes
Tigecycline Evaluation and Surveillance Trial (TEST), IHMA	2004 ongoing	Bacteria	Case-finding based on specimens sent to laboratory for clinical purposes
Typhoid Fever Surveillance in Africa Programme (TSAP), International Vaccine Institute, Korea	2009 ongoing	Bacteria	Case-finding based on specimens sent to laboratory for clinical purposes
WHO Western Pacific Regional Programme for Surveillance of Antimicrobial Resistance	1991—1998	Bacteria	Case-finding based on specimens sent to laboratory for clinical purposes
ZyvoxVR Annual Appraisal of Potency and Spectrum (ZAAPS), JMI Laboratories, USA and Pfizer	2004 ongoing	Bacteria	Case-finding based on specimens sent to laboratory for clinical purposes

a A tentative timeline (https://sedric.org.uk/amr-surveillance-projects/).
