# Supplementary material for: Surveillance strategies using routine microbiology for antimicrobial resistance in low- and middle-income countries
Source: Clin Microbiol Infect. Author manuscript; Available in PMC 2022 Sep 6. (PMC7613529; doi:10.1016/j.cmi.2021.05.037)
Supplement: Supplementary data [file EMS153535-supplement-Supplementary_data.docx]

**Supplementary Material**

**Search strategy**

**Query: What are the surveillance strategies used for antimicrobial resistance in low and middle-income countries?**

**For surveillance**

((epidemiology[MeSH Terms]) OR (public health surveillance[MeSH Terms]) OR (population surveillance[MeSH Terms]) OR (epidemiologic methods[MeSH Terms]) OR (surveillance[Title/Abstract]) OR (sampl*[Title/Abstract])) AND ((microbiology[Title/Abstract]) OR (microbiology[MeSH Terms]) OR (resistan*[Title/Abstract]))

**AND for microbiology**

((microbiology[Title/Abstract]) OR (microbiology[MeSH Terms]) OR (resistan*[Title/Abstract]))

**AND for low and middle-income countries**

((developing Countries[MeSH Terms]) OR (low resource[Title/Abstract]) OR (poor resource[Title/Abstract]) OR (limited resource[Title/Abstract]) OR (resource limited[Title/Abstract]) OR (LMIC[Title/Abstract]) OR (middle-income[Title/Abstract]) OR (Malawi[Title/Abstract]) OR (Democratic People’s Republic of Korea[Title/Abstract]) OR (Liberia[Title/Abstract]) OR (Gambia[Title/Abstract]) OR (Tanzania[Title/Abstract]) OR (Yemen[Title/Abstract]) OR (Afghanistan[Title/Abstract]) OR (Ethiopia[Title/Abstract]) OR (Madagascar[Title/Abstract]) OR (Mali[Title/Abstract]) OR (Nepal[Title/Abstract]) OR (Syrian Arab Republic[Title/Abstract]) OR (Uganda[Title/Abstract]) OR (Ghana[Title/Abstract]) OR (Mauritania[Title/Abstract]) OR (Bhutan[Title/Abstract]) OR (Cote d'ivoire[Title/Abstract]) OR (Kenya[Title/Abstract]) OR (Jerusalem[Title/Abstract]) OR (Occupied Palestinian Territory[Title/Abstract]) OR (Zambia[Title/Abstract]) OR (Zimbabwe[Title/Abstract]) OR (Bangladesh[Title/Abstract]) OR (Cambodia[Title/Abstract]) OR (Egypt[Title/Abstract]) OR (India[Title/Abstract]) OR (Indonesia[Title/Abstract]) OR (Jordan[Title/Abstract]) OR (Laos[Title/Abstract]) OR (Lao People’s Democratic Republic[Title/Abstract]) OR (Mozambique[Title/Abstract]) OR (Myanmar[Title/Abstract]) OR (Nigeria[Title/Abstract]) OR (Pakistan[Title/Abstract]) OR (Philippines[Title/Abstract]) OR (Sri Lanka[Title/Abstract]) OR (Sudan[Title/Abstract]) OR (Tunisia[Title/Abstract]) OR (Gabon[Title/Abstract]) OR (Libya[Title/Abstract]) OR (Mauritius[Title/Abstract]) OR (Bosnia and Herzegovina[Title/Abstract]) OR (Brazil[Title/Abstract]) OR (Georgia[Title/Abstract]) OR (Iran[Title/Abstract]) OR (Iraq[Title/Abstract]) OR (Lebanon[Title/Abstract]) OR (Malaysia[Title/Abstract]) OR (Maldives[Title/Abstract]) OR (Russia[Title/Abstract]) OR (South Africa[Title/Abstract]) OR (Thailand[Title/Abstract]) OR (North Macedonia[Title/Abstract]) OR (Viet*[Title/Abstract]))

((viral[Title/Abstract]) OR (fungal[Title/Abstract]) OR (parasite[Title/Abstract]) OR (plant[Title/Abstract]) OR (animal[Title/Abstract]) OR (livestock[Title/Abstract]) OR (food[Title/Abstract]) OR (gene*[Title/Abstract]) OR (sequencing*[Title/Abstract]) OR (water[Title/Abstract]) OR (tuberculo*[Title/Abstract]) OR (malaria[Title/Abstract]) OR (HIV[Title/Abstract]) OR (coloni*[Title/Abstract]))

AND (humans[Filter])

**Search was performed on 8th January 2021**
